# Editorial: Gene therapy for hearing loss: from mechanism to clinic

**DOI:** 10.3389/fnins.2023.1235627

**Published:** 2023-07-12

**Authors:** Shengyu Zou, Wenwen Liu, Yu Sun, Qingyin Zheng, Zuhong He

**Affiliations:** ^1^Department of Otorhinolaryngology-Head and Neck Surgery, Zhongnan Hospital of Wuhan University, Wuhan, China; ^2^Department of Otolaryngology-Head and Neck Surgery, Shandong Provincial ENT Hospital, Shandong University, Jinan, China; ^3^Department of Otorhinolaryngology, Union Hospital, Tongji Medical College, Huazhong University of Science and Technology, Wuhan, China; ^4^Department of Otolaryngology-Head & Neck Surgery, Case Western Reserve University, Cleveland, OH, United States

**Keywords:** sensorineural hearing loss, hair cell, auditory nerve, hearing regeneration, gene therapy

As the most prevalent sensory disorder, hearing loss causes patients a great deal of hardship and leads to a number of psychological and mental disorders. Such as the elderly individuals with presbycusis will causing social isolation, depressive symptoms and mild cognitive impairment. In addition, the cochlear hair cell loss and the degradation of auditory cortex are typical pathological alterations that lead to presbycusis. As pathological changes occur during the process of hearing loss, cognitive abilities and sound perception can be affected by lesions in the cochlea, auditory nerve, or auditory center. The common factors that can cause sensorineural hearing loss include genetic factors, medicines, infections, diseases, trauma, age, and the environment. With in-depth investigation of the pathophysiology of hearing disorders and enhancement of diagnosis and treatment technologies, gene therapy in hearing regeneration is gradually attracting our interest and is a future exploration direction. Gene therapy is a novel approach to preventing hair cell death, promoting hair cell regeneration and regulating the expression of genes associated with deafness. Remarkable advances have been made in gene therapy for hearing loss, but there are still many obstacles to overcome. New technologies and material applications will help us better understand the process of hearing loss and optimize the impact of gene therapy to restore auditory function. This Frontiers Research Topic entitled “*Gene therapy for hearing loss: from mechanism to clinic*” encompasses 11 manuscripts on the topic of gene therapy for sensorineural hearing loss, inner ear development regulation and clinical surgery treatment.

In the cochlea, inner ear cells are in charge of receiving acoustic signals and transmitting auditory information to the brain. Ribbon synapses can facilitate the conveying of signals to the appropriate spiral ganglion neurons by inner ear cells. However, hidden hearing loss (HHL) caused by ribbon synapse loss is usually ignored, and the mechanisms underlying ribbon synapse loss are largely unknown. Hou et al. conducted a series of experiments using an FGF22 knockout mouse model (*Fgf*22^−/−^) to explore the relationship between the *Fgf22* gene and synaptic defects in HHL. The authors found that *Fgf*22^−/−^ mice had lower wave I amplitudes at 8 and 16 kHz than *Fgf*22^+/+^ mice. However, the numbers of inner hair cells and ribbon synapses in *Fgf*22^−/−^ mice was not significantly different from those in *Fgf*22^+/+^ mice. The authors found that FGF22 deletion may cause calcium channels to be more easily activated and cause Ca^2+^-triggered exocytosis in response to stimuli. The real-time PCR results showed that the synaptic vesicle-related proteins SNAP-25 and Gipc3 were significantly decreased in *Fgf*22^−/−^ mice, which may affect the function of IHC ribbon synapses and induce HHL.

Endoplasmic reticulum (ER) stress is well-investigated in aminoglycoside-induced hearing loss, and ER stress inhibition exhibits a protective effect on aminoglycoside-induced ototoxicity. Wu et al. devoted much work to the protective role of pitavastatin (PTV), a new-generation lipophilic statin, on neomycin-induced hair cell loss. In *in-vitro* experiments, PTV protected not only HEI-OC1 cells but also cochlear explant cultures from hair cell damage caused by neomycin. In a mouse model, pretreatment with intraperitoneal injection of PTV prevented neomycin-induced hearing loss and hair cell damage. The authors further explored the mechanism underlying the protective effect of PTV and found that PTV could inhibit neomycin-induced apoptosis and ES under neomycin stimulation. In addition, PTV could inhibit PERK/eIF2α/ATF4 signaling to attenuate ER stress and inhibit the Rho/ROCK signaling pathway to protect against neomycin-induced ototoxicity.

Chen P. et al. reviewed the pathological mechanisms of connexin-26 (Cx26)-related hearing loss. Members of the Cx family play important roles in the cochlea, as these proteins can form gap junctions, which maintain the balance of ion and substance exchange between cells. The authors summarized two types of Cx26 transgenic mice, congenital deafness and late-onset progressive deafness model mice, and described the different pathological changes of these two models. Cxs are crucial for gap junction (GJ) combination, and Cx26 mutation disrupts the GJ system, which maintains stable endocochlear potential. The authors further explored the hypothesis that Cx26 deficiency causes cochlear potassium recycling dysfunction and put forth his own perspective. Furthermore, the authors discussed the crucial regulatory functions of Cx26 in the Ca^2+^ signaling pathway throughout the development of the inner ear. GJs play an important role in energy supply and glucose transport in the inner ear during development and hair cell (HC) survival. Mutations in Cx26 cause energy supply limitations and lead to Corti development disorder, OHC function decline and hearing failure.

Fei et al. introduced semicircular canal occlusion (SCO) to the treatment of vertigo in clinical and related basic research. SCO was initially applied to treat benign paroxysmal positional vertigo (BPPV). Later, semicircular canal fistula and Meniere's disease were also treated with SCO. The vertigo of most patients with Meniere's disease is controlled vertigo after SCO. The efficiency and safety of TSCO in the ear with endolymphatic hydrops were confirmed in animal models. Some patients with refractory BVVP who undergo posterior SCO show complete resolution of positional vertigo after long-term follow-up. However, some patients experience short-term hearing loss, which can recover within 6 months. Superior SCO is an ideal operation for treating superior semicircular canal dehiscence syndrome and has a low incidence of complications. Although the vertigo of most patients who undergo SCO is alleviated, there are still a few reports of hearing deterioration after SCO. Hence, further exploration is needed to determine the effect of SCO on hearing and vestibular function.

Adeno-associated virus (AAV) is a widely used vector for gene therapy in animal models and clinical trials. However, due to its packaging capacity, AAV cannot deliver a large gene or multiple genes. Therefore, a procedure for the cotransduction of dual-AAV vectors has been developed to solve these problems. Chen Z.-R. et al. tested the efficiency and safety profiles of cotransduced dual-AAV vectors in the vestibular sensory epithelium of mice. First, mixtures of dual-AAV-ie vectors with CGA or CMV promoters were injected into the adult and neonatal mouse inner ear. Compared with adult mice, the cotransduction rates in the striolar and extrastriolar region hair cells were significantly higher in neonatal mice. After diphtheria toxin administration to damage the utricle HCs, the authors found that the cotransduction rate increased in striolar and extrastriolar region HCs compared with those of control adult mice. Three months after dual-AAV-ie vector transduction, mouse auditory and vestibular functions were tested, and no significant effects were observed. The cotransduction efficiency was also maintained for 3 months in adult mice. This study provided a feasible safety profile for future gene therapies through the cotransduction of dual-AAV vectors into the vestibule.

G protein-coupled receptors (GPCRs) are the largest superfamily of mammalian cell surface receptors, and some of them are related to hearing disorders. Ma et al. reviewed 53 GPCRs expressed in the cochlea and offered a new GPCR-based medication development strategy for hearing loss therapy. In the manuscript, class A, class B2, class C and class F GPCRs in the cochlea and their functions are described. With the development of structural analysis, researchers have designed several GPCR structure-oriented drugs that target the complicated structure of GPCRs. With the exploration of cochlear gene therapy and GPCR-related gene therapy drugs that are applied in the treatment of other organ diseases, it is hoped that inner ear-oriented GPCR-related drugs will emerge in the near future.

Cochlear ribbon synapse maturation in postnatal mice requires morphological and functional modifications. Song et al. observed IHC ribbon synapse changes from P1-P28, and the data showed that the number of synapses undergoing pruning was accompanied by the activation and migration of macrophages. The expression of CX3CR1 was significantly associated with macrophage activation, and the expression level was increased at P7 and gradually decreased thereafter. Moreover, consecutive injection of a CX3CR1 inhibitor for 7 days after P7 showed that synapse pruning was affected, with impaired hearing in adulthood. Spiral neurons secreted CX3CL1 to recruit migrating macrophages at postnatal day 7. Exogenous supplementation with CX3CL1 activated synapse pruning and reduced the number of IHC ribbon synapses, and hearing was also impaired at P28. These results indicate that ribbon synaptic remodeling by macrophages via the CX3CL1/CX3CR1 axis occurs postnatally in mice prior to the onset of hearing and that this remodeling is essential for the development of hearing.

As a transient cellular cluster structure, Kölliker's organ (KO) plays a crucial role in cochlear development and degenerates during cochlear maturation. Chen J. et al. reviewed the morphological changes, biological functions and potential mechanisms for HC regeneration in KO. The structure of KO-SCs folds, cell gaps widen, and columnar cell numbers decrease and are replaced by cuboidal-like cells during maturation. The rhythmic morphological changes may be triggered by the intracellular Ca^2+^ concentration and contractile protein activation. Spontaneous ATP and Ca^2+^ release from KO-SCs is crucial for cochlear development, and KO-SCs secrete glycoproteins for tectorial membrane formation. Chen J. et al. also described the regulatory role of the apoptosis pathway, thyroid hormone signaling and autophagy in KO-SC programmed degeneration. Through the regulation of a variety of transcription factors and signaling pathways, KO-SCs can be a source of progenitor cells for hair cell regeneration in the early postnatal period, providing opportunities for the treatment of hearing disorders in the future.

Meng et al. compared the changes in vestibular function and hearing after different semicircular canal surgery techniques were performed for labyrinthine fistula treatment. In Group 1, the labyrinthine fistula was only partially covered with simple fascia when dexamethasone injection was administered. In Group 2, the labyrinthine fistula was capped with a “sandwich” composed of fascia, bone meal, and fascia. After surgery, several Group 1 patients experienced vertigo symptoms. The patients in Group 2 with type II labyrinthine fistulas showed short-term vertigo symptoms after surgery but no occurrences of vertigo during a long-term follow-up. For the patients with type I labyrinthine fistulas, there was no significant difference between bone conduction (BC) and air conduction (AC) thresholds or the A-B gap after surgery. However, for patients with type II labyrinthine fistulas, the recovery of the A-B gap in Group 2 was poorer than that in Group 1, but there was a significant recovery in hearing compared to preoperative hearing.

Mu et al. explored FOXG1-related epigenetic modifications that affect HC survival in cisplatin treatment. The expression of FOXG1 and the autophagy pathway were initially activated in both *in vivo* and *in vitro* models after cisplatin administration, and the expression decreased at high doses of cisplatin treatment. Subsequently, the authors found that the inhibition of FOXG1 and the autophagy pathway might be regulated by H3K9 methylation. Pretreatment with BIX01294, a G9a inhibitor that could transiently and reversibly inhibit H3K9me2 activity, reverse the inhibition of FOXG1 and autophagy and alleviate the HC damage and hearing loss induced by cisplatin. Interestingly, the authors also found that FOXG1 and H3K9me2 could affect the activation of the autophagy pathway by regulating autophagy-related miRNAs and affecting the ROS levels and apoptosis ratio changes in OC1 cells after cisplatin treatment.

Sun et al. provided a review of recent research progress in the role and regulation of autophagy and ferroptosis in the inner ear. As an important physiological process for metabolic waste degradation and reuse, autophagy plays a key role in maintaining normal cellular functions. In the auditory system, autophagy activation mostly promotes HC survival, such as in aging, ototoxic drug use and noise-related hearing loss. The autophagy pathway is regulated by many factors and signals, and this pathway needs to be further explored for deafness prevention. The authors also described ferroptosis regulation mechanisms in the auditory system and demonstrated that the prevention of ferroptosis activation had a protective effect on HCs during ototoxic drug exposure and aging-induced auditory cortex degeneration. Ferritinophagy, autophagy-induced ferroptosis, has recently attracted much attention from many researchers. Ferritinophagy is a kind of selective autophagy process that is mediated by NCOA4. NCOA4 induces the binding of autophagosomes and ferritin, and free Fe^2+^ increases with the degradation of ferritin and induces ferroptosis in cells. More studies are needed to clarify the mechanisms of ferritinophagy in the auditory system.

For this Research Topic, research on inner ear hair cell protection, ribbon synapses and organ of Corti development regulation and clinical middle ear surgery was collected. The content of this Research Topic includes recent research progress in the auditory system and the latest inner ear gene therapy methods and thus could provide a reference for further exploration of auditory disorders.

## Author contributions

SZ and ZH participated in writing the manuscript. ZH, SZ, WL, YS, and QZ participated in reviewing the manuscript. All authors contributed to the article and approved the submitted version.

